# Editorial: Disruptions by COVID-19: experiences of global health interventions in research, education, and humanitarian work

**DOI:** 10.3389/fsoc.2023.1102203

**Published:** 2023-07-28

**Authors:** Martha Judith Chinouya, Sepeedeh Saleh, Beth Maina-Ahlberg

**Affiliations:** ^1^Liverpool School of Tropical Medicine, Faculty of Education, Liverpool, United Kingdom; ^2^Department of Sociology, Uppsala University, Uppsala, Sweden; ^3^The Skaraborg Institute for Development, Skovde, Sweden

**Keywords:** COVID-19, global health, education, research, ethics, care, decolonizing, Ubuntu

With the advent of the COVID pandemic, restrictions on international and local travel and on human contact were imposed to limit the spread of the infection. This disrupted travel and physical human contact, aspects of global health practice, research, education, and humanitarian work, which have always been taken for granted. The articles in this Research Topic illuminate how the “disruptions” brought about by the COVID-19 pandemic to global health practices were experienced. The pausing of international travel, together with the restructuring of teaching and research to online, offered reflective opportunities to explore, what we thought at the time, were “disruptions” by COVID-19 and how these were experienced by practitioners, communities, and students. The submitted papers reveal the complex nature of these (dis)ruptions' as COVID resulted in changes in practices, but also in “busting open” new ways of knowing and experiencing global health, with the remodifications offering “new” continuation of global health practices from the discipline's colonial cradle.

The four contributions to the Research Topic include a reflection from a transnational public health doctor on her “Shifting Positionalities” during the first wave of COVID-19. Before the pandemic, she had as a student embarked on an ethnography of smoke in a rural village in Malawi. The pandemic resulted in turning her into a public health practitioner, doing health promotion work, providing resources to villagers for mitigating the spread of the COVID (Saleh et al.). Students from a university in the United States of America reflected on how the pausing of travel had enabled them to experience a much broader and richer definition of global health as they embarked on research in their local contexts. Importantly the students recommend that there is need to reflect on and address recruitment processes that only favor global health research abroad (see Chu et al.). Such reflection and change can help reduce the carbon footprints of traveling to faraway lands to conduct “global health research”. As physical contact with non-household members was discouraged in order to prevent the spread of the infection, researchers in London, have written about their experiences of conducting research using remote interviews with Black African women as technology enabled them to conduct interviews on the telephone (see Madziva and Chinouya). In South Africa where nursing practices are also guided by Indigenous knowledge systems of Ubuntu, there were important questions raised about the universal pausing of hospital visits during the pandemic (see Mulaudzi et al.). Ubuntu, the art of being human and treating others humanely, is the African ethic that continues to be a resource in times of adversities including during hospitalization (O'Keefe and Chinouya, 2005).

These are diverse perspectives on COVID-related (dis)ruptions, emanating from different geographical spaces. As editors we ask ourselves: what have we learnt from these contributions as we live in a precarious world in which pandemics exist? What do these papers tell us about the broader contexts of global health? Students' reflections lead to questions about teaching, recruitment priorities of some global health programs and the potential of enriching student experience through broadening of the meaning of global health to include research in local contexts of high-income countries. The definition of global health research, whilst promoting research in low-income countries, has not promoted, in equal proportion, the flow of students from low income countries to study “exotic” experiences found in high income countries. In this way, global health research would further enhance the value of cross-cultural or collaborative studies.

Threads connecting these perspectives include concerns about the ethics of global health practices, that are underpinned by the power of Western epistemologies and the focus on individualism. The COVID-19 pandemic has put the spotlight on individuals, their complex identities and networks, all which must be considered in health care, education, research and humanitarian work. The definition of global health research, including humanitarian work should put more emphasis on fluidity of place of the interventions, to include local neighborhoods in high income countries as well as those “disadvantaged populations, in exotic ‘foreign' lands. The advancement in digital and mobile technology conceptually shifts our imagination of the ‘global' and local context of the disciplines” practices. The technological capabilities are allowing practitioners, researchers, and students to move forward with a “new COVID normalcy,” which is socially produced in different contexts. Through technology, there has been an increase in “remote working” or working from “home”, online learning, online interviews/research, and online medical appointments. All these practices, as highlighted in the articles in the Research Topic, contribute toward redefining meanings and our ways of understanding and experiencing “presence,” “space”, “human contact”, and “visits” as the world adapts to reduce risks of COVID due to human contacts during research, in the classroom, in hospitals and in communities in general. Importantly, indigenous knowledge systems remind us of the importance of negotiating cultures in health.

Whilst appearing as “new” interventions, some of these practices such as working from home, are not new. Before the industrial revolution, working from home was the norm, and in the 1980s, telecommunication technologies enabled work to be brought home replacing physical travel to a central office (Olson and Primps, 1984). The reflections above are particularly important in view of the impacts of the ‘lockdown', or the new normal of working from home, schooling from home, and reduced transport (including air travel), on carbon emissions, and consequent potentials for improved health (Cicala et al., 2021). However, we need to ask whether these positive changes can be maintained given that pandemics have always forced humans to break with the past and imagine their world anew. In conclusion we look back with nostalgia at the pre-COVID-19 pandemic world within the context of our experiences and ask ourselves if these were disruptions or mere continuities of previous disruptions from past epidemics.

## Author contributions

All authors listed have made a substantial, direct, and intellectual contribution to the work and approved it for publication.
